# Hypertriglyceridemic waist may explain ethnic differences in hypertension among patients with type 2 diabetes in Sweden

**DOI:** 10.1186/1756-0500-5-474

**Published:** 2012-08-31

**Authors:** Marina Taloyan, Nuha Saleh-Stattin, Sven-Erik Johansson, Lars Agréus, Per Wändell

**Affiliations:** 1 Karolinska Institutet, Centre for Family and Community Medicine, Alfred Nobels allé 12, Huddinge, SE, 141 83, Sweden; 2 Stress Research Institute, Stockholm University, Stockholm, Sweden; 3 Centre for Primary Health Care Research, Region Skåne, Lund University, Malmö, UMAS, 205 02, Sweden

**Keywords:** Ethnicity, Hypertension, Hypertriglyceridemic waist, Type 2 diabetes, Sweden

## Abstract

**Background:**

Hypertension is common among persons with type 2 diabetes. The aim of this study was to analyze the association between ethnicity and hypertension prevalence after adjusting for age, sex, Hba1c, total cholesterol, elevated triglycerides and hypertriglyceridemic waist. The study population consisted of 354 primary health care patients diagnosed with type 2 diabetes (173 Assyrians/Syrians and 181 Swedes) residing in Södertälje, Sweden. Unconditional logistic regression was used to analyze the data.

**Results:**

Hypertension prevalence was higher among Swedes than Assyrians/Syrians, (77% versus 58%; p = 0.001). In the unadjusted logistic regression model, the odds ratio for hypertension in Swedes was twice as high than that in Assyrians/Syrians (OR = 2.44; 95% CI =1.54-3.86). In the age- and sex-adjusted model, odds ratio of hypertension was 2.25 (95% CI 1.41-3.60). After adjustments for total cholesterol was made, the odds ratio of hypertension decreased slightly to 1.73. When elevated triglycerides and hypertriglyceridemic waist were separately introduced, the odds ratio of hypertension was no longer significant between the ethnic groups (1.60 and 1.43 for triglycerides and hypertriglyceridemic waist respectively). In addition, advanced age – 60–69 years old (OR = 1.80, CI 95% 1.00-3.20) and ≥ 70 years old (OR = 2.88, CI 95% 1.40-5.93), elevated total cholesterol (OR = 1.48, CI 95% 1.12-1.95) and presents of hypertriglyceridemic waist (those with high WC and high TG) were significant confounding factors for the increased risk of hypertension independent of ethnicity.

**Conclusions:**

The crude differences in prevalence of hypertension between the Swedes and Assyrians/Syrians in our study population with type 2 diabetes were no longer significant when adjusting for high triglycerides levels or the presence of hypertriglyceridemic waist.

## Background

Type 2 diabetes is an independent risk factor for cardiovascular disease [1] and diabetes-related complications [2]. Hypertension and hyperglycaemia are common in persons with type 2 diabetes and have been shown to increase the risk of cardiovascular disease [3,4]. Risk factors for developing hypertension include age, high alcohol intake [5] and dyslipidemia [6], smoking [7], stress [8], lifestyle [9], and family history of hypertension [10]. High body mass index (BMI) [11] and physical inactivity [12] are common risk factors for developing both hypertension and type 2 diabetes. Diabetes, hypertension, elevated triglycerides and hypertriglyceridemic waist (hyperTG waist) are all features of the metabolic syndrome [13].

Previous studies on the relationship between migration, ethnicity, hypertension, and diabetes have reported a variety of findings. For instance, a study from the U.S. showed that age and BMI, possibly combined with presence of a psychiatric disorder, history of trauma, and immigrant status, contributed to a higher overall prevalence of both hypertension and diabetes in immigrants than in native Americans [14]. By contrast, in Sweden, hypertension was shown to be less common in immigrants from non-European countries than in native Swedes after adjusting for BMI [15].

Few studies have looked into the prevalence of hypertension in immigrants with type 2 diabetes living in Sweden. Previous analyses of the data used in this study showed that Swedes with type 2 diabetes had a higher prevalence of and more twice as high odds of hypertension than had Assyrians/Syrians with type 2 diabetes. These differences between the groups could not be explained by differences in sociodemographic situation [16]. Hence, the aim of this study was to analyze the association between ethnicity and hypertension prevalence after adjusting for age, sex, anthropometric measures and blood lipids.

## Results

The study population consisted of 354 individuals with type 2 diabetes living in Södertälje, Southern Sweden, 173 of whom were Assyrians/Syrians and 181 were Swedes. Men and women were equally represented in the sample (Table 1). The Assyrians/Syrians had a mean age of 61 years (range 32–83 years), whereas Swedes had a mean age of 64 years (range 32–86 years). More than a third of the Swedes (35.0%) and less than a third of the Assyrians/Syrians (26.2%) were ≥ 70 years of age. Moreover, obesity (BMI ≥ 30) was more prevalent among Assyrians/Syrians (57.0%) than Swedes (42.0% *P* = 0.038), whereas an overweight was more prevalent among Swedes (44.5%) than Assyrians/Syrians (30.0%). Moderate self-reported alcohol consumption was higher among Swedes (81.0%) than Assyrians/Syrians (44.5%) and more than half of the Assyrians/Syrians (52.5%) and less than a fifth (15%) of the Swedes reported they had never used alcohol.

**Table 1:** Distribution (%) of background variables and anthropometric data (means; standard deviations) on Assyrian/Syrians and Swedes (n = 354)

**Variables**	**Assyrians/Syrians n = 173**	**Swedes n = 181**	**P value**
**Total**	48.9	51.1	
**Sex**			
Women	48.5	44.2	0.411
Men	51.5	55.8	
**Age (years)**			
32–59	46.5	32.6	0.026
60–69	27.3	32.6	
≥70	26.2	34.8	
**Body mass index**	33 (20.2)	30 (6.0)	0.059
**BMI (kg/m**^**2**^**)**			
Normal (<25)	13.0	13.5	0.038
Overweight (25.0–29.9)	30.0	44.5	
Obese (≥30)	57.0	42.0	
**Abdominal obesity**	106.1 (12.1)	106.3 (13.8)	0.877
Women (>88 cm)	62.0	52.0	0.205
Men (102 cm)	38.0	48.0	0.447
**Alcohol consumption**			
High	3.0	4.0	0.000*
Moderate	44.5	81.0	
Low	52.5	15.0	
**Medication** (yes)	78.1	78.6	0.912
**Elevated total cholesterol** (≥4.5 mmol/L)	59.0	65.0	0.359
**Elevated triglycerides** (≥1.7 mmol/L)	40.0	64.0	**0.000**
**Elevated HbA1c** (>6%)	49.7	40.6	0.088
**Hypertension**	58.0	77.0	**0.001**
**HyperTG waist†**			
Low WC + low TG	16.3	12.2	0.422
Low WC + high TG	9.9	14.9	
High WC + low TG	18.6	16.0	
High WC + high TG	55.2	56.9	

*Bold-faced numbers are statistically significant and corrected for multiple statistical testing (<=0.005).

† Low WC = ♂ ≤ 102 ♀ ≤ 88; Low TG = < 2.0; High WC = ♂ >102 ♀ >88 WC; High TG = ≥ 2.0.

The prevalence of elevated HbA1c, elevated total cholesterol, and elevated triglyceride levels in the two groups are shown in Table 1. There were no significant differences in the prevalence of either elevated HbA1c or total cholesterol levels, nor were there any differences in use of medication and presence of hyperTG waist, between the Assyrians/Syrians and Swedes. However, Swedes had a significantly higher prevalence of elevated triglycerides (64.0%) than had Assyrian/Syrians (40.0%) and prevalence of hypertension was significantly higher in Swedes (77.0%) than was in Assyrian/Syrians (58.0%).

Prevalence of hypertension by ethnicity and sex in the two groups is shown in Table 2. We found significant differences in alcohol consumption and elevated triglycerides between Swedish and Assyrian/Syrian women. The majority of Assyrian/Syrian women were low-level alcohol consumers, whereas most Swedish women were moderate alcohol consumers. Elevated triglycerides were significantly more prevalent in Swedish than in Assyrian/Syrian women. By contrast, no significant differences in either alcohol consumption or elevated triglyceride prevalence were found between Swedish and Assyrian/Syrian men.

**Table 2:** Prevalence (%) of hypertension and explanatory variables by ethnicity and sex

	**Women**		***P *value**	**Men**		***P *value**
	**Assyrian/Syrian**	**Swedes**		**Assyrian/Syrian**	**Swedes**	
	**n = 89**	**n = 101**		**n = 84**	**n = 80**	
**Variables**						
**Totals**	56.1	80.0	0.007*	59.1	74.0	0.065
**Body mass index**						
Normal <25	12.0	10.0	0.050	14.0	16.0	0.475
Overweight 25.0–29.9	23.0	44.0		37.0	45.0	
Obese ≥30	65.0	46.0		49.0	39.0	
**Abdominal obesity**						
Women (>88 cm)	93.5	88.5	0.383			
Men (102 cm)				53.7	62.8	0.295
**Alcohol consumption**						
High	0.0	0.0	<0.0001	6.0	7.2	0.271
Moderate	18.0	84.0		69.0	78.3	
Low	82.0	16.0		25.0	14.5	
**Medication** (yes)	81.1	82.3	0.876	77.8	75.9	0.828
**Elevated total cholesterol**						
(≥4.5 mmol/L)	65.2	70.0	0.582	53.5	61.0	0.380
**Elevated HbA1c** (>6%)	50.0	56.0	0.522	46.5	33.3	0.112
**Elevated triglycerides**						
(≥1.7 mmol/L)	41.0	74.0	<0.0001	38.0	56.5	0.028*
**HyperTG waist**†						
Low WC + low TG	34.8	21.0	0.094	5.0	2.1	0.091
Low WC + high TG	10.9	16.1		2.5	8.5	
High WC + low TG	43.4	35.5		57.5	34.1	
High WC + high TG	10.9	27.4		35.0	55.3	

* Statistically non-significant after correction for multiple statistical testing (<=0.007).

†Low WC = ♂ ≤ 102 ♀ ≤ 88; Low TG = < 2.0; High WC = ♂ >102 ♀ >88 WC; High TG = ≥ 2.0.

Table 3 shows the prevalence of hypertension by ethnicity and explanatory variables, such as age, sex, BMI, abdominal obesity, alcohol consumption, medication, elevated total cholesterol, elevated HbA1c, elevated triglycerides and presence of hyperTG waist. Mean systolic blood pressure reading was significantly higher in Swedes than was in Assyrians/Syrians. In addition, Swedes with elevated total cholesterol had an overall higher prevalence of hypertension than had Assyrian/Syrians with elevated total cholesterol.

**Table 3:** Prevalence (%) of hypertension (systolic ≥130 mm Hg or diastolic ≥ 80 mm Hg) and means with standard deviations (SD) blood pressure in Assyrians/Syrians and Swedes by ethnicity and explanatory variables and test of differences in distribution by ethnicity (chi2)

**Variables**	**Prevalence of hypertension**		
	**Swedish-born**	**Assyrian/Syrian-born**	***P *value**
**Totals**	77.0	58.0	0.001*
**Age**			
32–59	76.0	46.4	**0.004**
60–69	66.0	67.0	0.939
≥70	88.0	72.4	0.103
**Sex**			
Women	80.0	56.1	0.007
Men	74.0	60.0	0.065
**Body mass index**			
Normal <25	75.0	50.0	0.134
Overweight 25.0–29.9	74.0	51.2	0.025
Obese ≥ 30	80.0	63.0	0.039
**Abdominal obesity**			
Women (>88)	80.0	56.5	0.011
Men (>102)	79.0	60.5	0.079
**Alcohol consumption**			
High	100.0	75.0	0.236
Moderate	76.0	57.4	0.014
Low	72.2	57.4	0.237
**Medication** (yes)	79.3	79.6	0.960
**Elevated total cholesterol**			
(≥4.5 mmol/L)	83.1	62.0	**0.003**
**Elevated HbA1c**			
(>6%)	80.4	67.0	0.099
**Elevated triglycerides**			
(≥1.7 mmol/L)	80.3	70.4	0.192
**Systolic/diastolic** Mean (SD)	142.4 (19.0) / 82.0 (13.3)	135.1 (17.0)/78.6 (11.4)	**0.0002**/0.025
**HyperTG waist†**			
Low WC + low TG	16.2 (24)	21.2 (31)	**0.001**
Low WC + high TG	11.5 (17)	6.2 (9)	
High WC + low TG	37.2 (55)	54.1 (79)	
High WC + high TG	35.1 (52)	18.5 (27)	

*Bold-faced numbers are statistically significant and corrected for multiple statistical testing (<=0.005).

†Low WC = ♂ ≤ 102 ♀ ≤ 88; Low TG = < 2.0; High WC = ♂ >102 ♀ >88 WC; High TG = ≥ 2.0.

### Logistic regression

In the unadjusted logistic regression model, the OR of hypertension among Swedes was more than twice as high than in Assyrians/Syrians (OR = 2.44; 95% CI =1.54-3.86). In the logistic regression models applied to estimate the effect of statistically significant factors are shown in Table 4. Abdominal obesity, alcohol consumption, BMI, use of medication, and HbA1c were shown to be not statistically significant and were thus excluded. Models 1–3 were generated after adjustments for age, sex, and elevated total cholesterol were made. Models 3 and 4 were generated stepwise by separate analyses with elevated triglycerides and presence of hyperTG waist. Ethnic differences in the prevalence of hypertension were not significant. Significant confounders were age, elevated total cholesterol levels and presence of hyperTG waist (Model 4). Although the variable elevated triglycerides was not significant, it could explain ethnic differences in prevalence of hypertension. In addition, advanced age – 60–69 years old (OR = 1.80, CI 95% 1.00-3.20) and ≥ 70 years old (OR = 2.88, CI 95% 1.40-5.93), elevated total cholesterol (OR = 1.48, CI 95% 1.12-1.95) and presents of hypertriglyceridemic waist (those with high WC and high TG) were significant confounding factors for the increased risk of hypertension independent of ethnicity.

**Table 4:** Odds ratios with 95% confidence intervals of hypertension in Assyrians/Syrians and Swedes with type 2 diabetes in four logistic regression models

**Variable**	**Crude OR**	**Model 1: (age, sex)**	**Model 2: (+chol)**	**Model 3: (+chol, +trig)**	**Model 4: (+chol, +hyperTG)**
**Assyrians/Syrians**	Reference	Reference	Reference	Reference	Reference
**Swedes**	2.44*	**2.25**	**1.73**	1.60	1.43
	1.54-3.86	1.41-3.60	1.05-2.84	0.95-2.70	0.84-2.44
**Age groups (years)**					
32–59		Reference	Reference	Reference	Reference
60–69		1.61	**1.80**	**1.88**	**1.80**
		0.95-2.72	1.03-3.12	1.05-3.33	1.00-3.20
≥70		**2.53**	**2.40**	**3.00**	**2.88**
		1.40-4.80	1.21-4.64	1.45-6.10	1.40-5.93
**Sex**					
Men		Reference	Reference	Reference	Reference
Women		1.20	1.43	1.20	1.42
		0.75-1.90	0.87-2.32	0.70-2.00	0.79-2.60
**Elevated total cholesterol**					
No			Reference	Reference	Reference
Yes			**1.50**	**1.44**	**1.48**
			1.16-1.96	1.10-1.90	1.12-1.95
**Elevated triglycerides**					
No				Reference	
Yes				1.30	
				0.96-1.70	
**HyperTG waist**					
Low WC + low TG					Reference
Low WC + high TG					2.50
					0.82-7.61
High WC + low TG					1.30
					0.62-2.70
High WC + high TG					**2.83**
					1.20-6.71

* Bold-faced numbers are statistically significant.

Abdominal obesity, alcohol consumption, medication and HbA1c were tested but found to be non-significant and therefore are not shown in the table.

## Discussion

### Summary of results and comparison with results from other studies

The main finding of this study was that ethnic differences in hypertension prevalence between Swedes and Assyrians/Syrians were no longer significant when adjustments for both elevated level of triglycerides and hyperTG waist were made. Furthermore, regardless of ethnicity, those with high WC and high TG had approximately three times higher odds of hypertension than those with low WC and low TG.

Our finding that Swedes had a higher prevalence of hypertension than Assyrians/Syrians had is not consistent with findings of a meta-analysis of 125 studies which showed an association between acculturation to Western society and high blood pressure. The authors of this meta-analysis concluded that acculturation to Western society was associated with increased blood pressure due to distress caused by cultural changes, independent of BMI or elevated total cholesterol [17]. However, a Swedish study of non-European immigrants showed a low risk of hypertension when adjustments for socio-economic, lifestyle and anthropometric factors were made [16].

In this study, we compared the prevalence of normal blood pressure among our participants with that of all persons included in the Swedish National Diabetes Register (NDR), a national quality-of-care register that covers almost 60 percent of all people in Sweden with diabetes and contains no information on ethnicity. NDR data from 2008 [18] indicate that 35.0% of the population in the register had normal blood pressure (≤130/80 mmHg). According to the data in this study, almost a fourth of the Swedes (23.3%) and nearly half the Assyrians/Syrians (42.1%) had normal blood pressure which suggests that the Swedes in our study population had a higher prevalence of hypertension (77%) than the national average (65%), whereas Assyrians/Syrians have a lower prevalence of hypertension (58%) than the national average.

In this study we did not have data on HDL-cholesterol levels and, thus, could not estimate the prevalence of metabolic syndrome among our participants. For that, we used the presence of hyperTG waist as a proxy for metabolic syndrome. However, we did not use the established definition of hyperTG waist for this population sample, i.e. waist circumference (WC) of >90 cm in men and >85 cm in women, as all men had a waist circumference larger than that, why we used another cut-off value (≤102) instead. As expected, we found that presence of hyperTG waist, namely the fourth group of hyperTG – high WC and high TG, comprised a significant explanatory factor for the prevalence of hypertension both among Assyrians/Syrians and Swedes. Given that diabetes, hypertension, elevated triglycerides and hypertriglyceridemi have all been shown to be features of metabolic syndrome, this finding was not unexpected. Another potentially important factor for hypertension prevalence was high alcohol intake, but very few of the participants in this study were high-level alcohol consumers, thus, the association between high alcohol intake and hypertension prevalence could not to be assessed. Besides, since this study included only individuals with type 2 diabetes, a putative association between alcohol intake and hypertension prevalence may be different than that among individuals without diabetes.

In this study we showed that Swedes had a higher prevalence of elevated triglycerides than had Assyrians/Syrians, which is not consistent with findings from a Swedish study of 60-year-old persons residing in Stockholm County [19]. In that study, non-European immigrants had higher ORs of high triglyceride levels and low HDL-cholesterol levels than did Swedish-born persons. However, our study included persons with type 2 diabetes, whereas the previous study included non-diabetic persons. Moreover, we only had access to data on total cholesterol levels which was why we could not analyze ORs of low HDL- or high LDL-cholesterol levels.

Studies have indicated that lifestyle choices such as exercise and low-sodium high-potassium diet can lead to decreased blood pressure [20] and increased carbohydrate intake was shown to be associated with elevated triglyceride levels [21]. However, we did not have access to data on dietary habits of the participants in in this study.

### Strengths and limitations of the study

A major strength of this study was its use of data from an ethnically homogeneous group, who identified themselves on an ethnic rather than a non-ethnic basis, i.e. as Assyrians/Syrians and not as nationals of their country or region of birth (Turkey, Lebanon, Middle East, etc). Despite the small sample size, the study population was considered representative of Assyrians/Syrians of four different countries who presently reside in Sweden. Recruiting participants from the same primary health care centers eliminated the possibility that factors such as environment and neighborhood, which have been shown to be significant confounders for the prevalence of hypertension, could impact on the results of the analyses. Although the findings of this study could not be generalized to all Assyrians/Syrians and Swedes with diabetes in Sweden, they may offer an avenue for future epidemiological studies.

The major limitations of the current study were the cross-sectional design, which precluded causal associations, and the small sample size. Due to the lack of data on HDL-cholesterol levels and medication prescribed to treat hypertension, we were not able to confirm presence of the metabolic syndrome. In addition, lack of information on whether hypertension was treated or not, meant that we could not draw any conclusions based on our results. To counteract this shortcoming, blood pressure measurements were performed.

## Conclusions

In conclusion, we found that the Swedes in our study population had a higher prevalence of hypertension than had the Assyrians/Syrians in the same population. The crude differences in prevalence of hypertension between the Swedes and Assyrians/Syrians in our study population with type 2 diabetes were no longer significant when adjusting for high triglycerides levels or the presence of hypertriglyceridemic waist.

## Methods

### Setting and participants

This cross-sectional study was based on a health survey conducted at four primary health care centers in Södertälje from 2006 to 2008 as the same study previously published in BMC Public Health [22]. One specific group, Assyrians/Syrians, was chosen for study because they are concentrated in great numbers in a single urban area located just south of Stockholm, the town of Södertälje, in which first and second generation immigrants (not all Assyrians/Syrians) constitute 44.0% of the total population according to statistics of Stockholm County. The registers over diabetes patients (already diagnosed by GP) at each primary health care centers were used.

### Data sources

Information on ethnicity, age, sex, and alcohol consumption was gathered during face-to-face interviews. Blood pressure and anthropometric measurements, including height, weight, and waist circumference, were made after the interview by the first author or participating GPs. Medical information and laboratory data were gathered from patient records after obtaining verbal informed consent from all participants. Patients with diagnosed type 2 diabetes were included in the survey.

### Explanatory variables

*Ethnicity* was defined as Swedish or Assyrian/Syrian. At the interview, all participants who had been provisionally identified as Swedes were asked “Are you Swedish?” Those who answered “No” were asked additional questions about country of birth and ethnic identity, and if they did not identify themselves as Assyrians/Syrians, were excluded from the study. Participants who were provisionally identified as Assyrians/Syrians were asked about country of birth and ethnic identity.

*Age* was stratified into three groups: 32–59, 60–69, and ≥70 years.

#### Anthropometric measurements

*Body mass index* (BMI) was calculated as weight divided by height squared (kg/m^2^). BMI < 25 was considered normal; BMI 25.0–29.9, overweight, and BMI ≥ 30, obese.

*Abdominal obesity* was defined as a waist circumference of ≥ 102 cm in men and ≥ 88 cm in women. Both *BMI* and *Abdominal obesity* were defined on the basis of established guidelines for adults [23].

Hypertriglyceridemic waist (HyperTG waist) is a suggested as a screening tool for identification of metabolic syndrome and based on several studies [24-26]. We choose to use HyperTG waist because of lack of data on HDL-cholesterol. Originally, a high waist circumference (WC) was defined by >90 cm in men and >85 cm in women, and a high triglycerides (TG) ≥2.0 mmol/l. However, as in our study no men showed a WC ≤90 cm we decided to use other cut-off values, i.e. >102 cm in men and 88 cm in women, often used in different definitions of the metabolic syndrome [13]. HyperTG waist was categorized into four groups: (1) low WC and low TG; (2) low WC and high TG; (3) high WC and low TG; (4) high WC and high TG.

#### *Alcohol consumption* was based on two questions on frequencies and quantities of drinking

– −Do you drink alcohol? Response alternatives were “often,” “sometimes,” and “never.”

– −Do you drink at least half a bottle of spirits or a couple of bottles of wine per week? Response alternatives were “often,” “sometimes,” and “never.” Participants who answered “never” in response to both questions were defined as low consumers. Those who responded that they often or sometimes drank alcohol and often drank at least half a bottle of spirits or a couple of bottles of wine per week were defined as high consumers. The rest were defined as moderate consumers. Categorization of alcohol consumption was based on validation done by Theobald [27].

*Medication* was based on response of taking drugs for normalization of HbA1c level or dietary treatment. We lack the information on medication against hypertension.

*Total cholesterol* was divided into two groups: normal (<4.5 mmol/L) and elevated (≥4.5 mmol/L).

*HbA1c* was divided into two groups: normal (≤6.0%) and elevated (>6.0%) using the Swedish mono-S method [28]. The mono-S method is the standard method in Sweden for defining normal and elevated HbA1c. Values ≥ 6% are considered elevated, which differs from the Diabetes Control and Complications Trial (DCCT) standard of >7%, which is the world standard [28].

*Triglycerides* were divided into normal (<1.7 mmol/L) and elevated (≥1.7 mmol/L).

All medical values were taken from patient journal records which were based on biochemical analyses performed at the same place at the laboratory at the Karolinska Universitet. According to the laboratory was HbA1c measured, after a chromatographic separation, through detection of absorbance at 415 nm by ”The Variant II Turbo Clinical Data Management (CDM) Software”, delivered by Bio-Rad, Variant II Turbo HbA1c Kit – 2.0 (Cat. No.270-2455EX). Total cholesterol was measured through detection of a colored quinoeimin product, after hydrolysis of cholesterol ester into free cholesterol, which then was oxidized to cholesten-3-one and hydrogen peroxide, the latter reacting with 4-aminantipyrin and phenol to the colored quinoeimin product, by DXC800 (2020) from Beckman-Coulter. Triglycerides were hydrolyzed to glycerol and free fatty acids, glycerol was reacted into glycerol-3-phosphate, oxidized into hydrogen peroxide, coupling 3,5-dichloro-2-hydroxybenzenesulfonic acid (DHBS) with 4-aminoantipyrine into a red complex which was measured, by DXC800 (2020) from Beckman-Coulter.

### Outcome variable

*Hypertension* was defined on the basis of the International Diabetes Federations guidelines for individuals with type 2 diabetes [29]. Blood pressure was measured once in the right arm in the sitting or lying position following 5 minutes of rest after the interview. The instrument used was a Boso Medicus Prestige Digital Sphygmomanometer; two different adult cuff sizes were used. Systolic blood pressure was dichotomized as normal (<130 mmHg) and high (≥ 130 mmHg). Diastolic blood pressure was divided into normal (<80 mmHg) and high (≥80 mmHg). If either the systolic blood pressure was ≥130 mmHg and/or the diastolic blood pressure was ≥ 80 mmHg, the patient was considered to be hypertensive.

### Statistical analyses

The description of the distribution of a number of the background variables included mean ± standard deviation. The prevalence, in percentage, of the dependent variable was estimated separately for Assyrians/Syrians and Swedes using the statistical software program Stata v.9 [30]. The test of level of significance in the prevalence of hypertension and all explanatory variables were performed by using Pearson’s chi-square test. Statistical significance was established at P < 0.05 (two-tailed P values). Unconditional logistic regression was used to calculate the ORs and 95% confidence intervals (95% CIs) to analyze the association between hypertension and explanatory variables. We used different models with different explanatory variables. In the second model, beside age and sex in addition to ethnicity, we tested elevated total cholesterol. In the third model elevated triglycerides and in the fourth model hyperTG waist was separately introduced. All medical values total cholesterol and triglycerides were included into logistic regression as continuous variables. High BMI, abdominal obesity, alcohol consumption, medication, and HbA1c did not reach significance and were excluded. The fit of the models was assessed by the Hosmer-Lemeshow goodness-of-fit test. This test is a Chi-square statistic from g*2 table (with g-2 degrees of freedom) of observed and expected frequencies [31]. The final models in logistic regression analyses were considered acceptable if p > 0.05, and all models met this demand [31].

### Ethical considerations

The study was approved by the Regional Ethical Committee of Karolinska Institutet (reference no. 2006/4:8, 2006-09-27). All participants received information about the study in written and verbal form. Participants provided verbal informed consent prior to the onset of participation and again before information was gathered from medical records.

## Abbreviations

BMI: Body mass index; CI: Confidence Interval; GP: General practitioner; HbA1c: haemoglobin A1c; HyperTG waist: Hypertriglyceridemic waist; NDR: National Diabetes Register; OR: Odds ratio; PHCC: Primary health care center; TG: Triglycerides; WC: Waist circumference.

## Competing interests

The authors have not declared any conflicts of interest.

## Authors’ contributions

MT conceived the idea for the survey. MT, NSS, SEJ, LA and PW designed the study. MT and PW performed the statistical analysis. MT drafted the manuscript. NSS, SEJ, LA and PW revised the manuscript. All authors read and approved the final manuscript.
